# Challenge or Hindrance? The Dual Impact of Algorithmic Control on Gig Workers’ Prosocial Service Behaviors

**DOI:** 10.3390/bs14060497

**Published:** 2024-06-14

**Authors:** Xuedong Liang, Wanting Fu, Peng Luo, Yanda Huo

**Affiliations:** Business School, Sichuan University, Chengdu 610064, China; liangxuedong@scu.edu.cn (X.L.); fuwanting0403@stu.scu.edu.cn (W.F.)

**Keywords:** algorithmic control, gig economy, affective events theory, prosocial service behaviors, challenge–hindrance appraisal framework, workplace interpersonal capitalization

## Abstract

Algorithmic technological progress presents both opportunities and challenges for organizational management. The success of online labor platforms hinges on algorithmic control, making it imperative to explore how this control affects gig workers’ prosocial service behaviors. Drawing from affective event theory, our study delves into the factors influencing gig workers’ prosocial service behaviors in the online labor platform setting. We utilize the challenge–hindrance appraisal framework to highlight the pivotal role of algorithmic control. To rigorously test our hypotheses, we gathered empirical data from an online questionnaire survey of 660 gig workers. Our results indicate that challenge appraisals and hindrance appraisals in regard to platform algorithm control have a nuanced dual impact on gig workers’ prosocial service behaviors. This relationship is clarified by the mediating function of work engagement. A challenge appraisal of platform algorithmic control can positively influence gig workers’ prosocial service behaviors. However, hindrance appraisal of platform algorithmic control can negatively influence gig workers’ prosocial service behaviors. Interestingly, workplace interpersonal capitalization boosts the effect of challenge appraisal on employees’ prosocial service behaviors. However, it does not mitigate the adverse effects of hindrance appraisal on such behaviors. This study has multiple theoretical implications, and it also provides valuable practical insights into organizational management.

## 1. Introduction

In the paradigm of the gig economy, online labor platforms, contrasted with conventional organizational management models, have innovated management practices by virtually supervising platform workers who are exempt from direct organizational oversight [1,2]. Specifically, digital technologies transcribe the labor processes of gig workers into a computationally analyzable data format. The labor data, acquired in real-time, are subjected to big data analytics and algorithmic predictions to achieve an optimal allocation of labor processes for each worker [3], a phenomenon identified as algorithmic control. As a convergent force of digital technologies and the gig economy, online labor markets have witnessed the pervasive implementation of algorithmic control [4], facilitating gig workers in attaining heightened output, enabling online labor platforms to maximize economic efficiency, and ensuring customers are recipients of superior service [5].

While the existing literature has established a preliminary foundation for ensuing in-depth exploration of the influential mechanisms underpinning algorithmic control, several theoretical vacuums remain to be substantively addressed. First, the extant literature presents a paradox concerning the impact of algorithmic control on employees with the concurrent existence of both promotional [6] and inhibitory effects [7]. While Cram et al. [8] and Liu et al. [9] have recognized this paradox, engaging in empirical research, their research models have predominantly concentrated on the ambivalent effects engendered by algorithmic control without isolating the binary nature of algorithmic control from its origin. Secondly, diverging from traditional organizations, the gig economy, epitomized by online ride-hailing platforms, necessitates employees to proffer exemplary service to passengers [10]. Nonetheless, a research void persists regarding the performance of gig workers’ service behavior within the framework of algorithmic control. Lastly, a discernible emphasis has been placed upon the nature of algorithmic control and the emotional state of workers in its impact process, with limited studies directing focus toward the impact of external social influences upon workers, transcending algorithmic control.

To address the posited research questions and ameliorate the identified research gap, this study strategically amalgamates the affective events theory with the challenge–hindrance appraisal framework (CHAF) and the conceptualization of prosocial service behaviors (PSBs), thereby guiding the formulation of the theoretical model. In examining the impact of challenge–hindrance appraisals on PSBs, work engagement (WE) is selected as the mediator, given its previously established status as an antecedent of PSBs and an outcome of challenge–hindrance appraisals [11,12], thereby showcasing its appropriateness as a mediator between challenge–hindrance appraisals and PSBs. Moreover, delving into potential contingencies that shape the relationship between multifarious factors and gig workers’ behaviors is imperative. Thus, this research endeavors to scrutinize the moderating impact of workplace interpersonal capitalization (WIC) on the relationships among work engagement, challenge–hindrance appraisals and PSBs.

The contributions of our research serve to augment the existing body of literature by introducing empirical insights and methodological advancements pertinent to the discussion of algorithmic control and worker behaviors within the gig economy. Firstly, it explores the positive and negative effects of algorithmic control on gig workers by incorporating challenge and hindrance appraisals to elucidate the underlying mechanisms of algorithmic control. Secondly, it enhances our understanding of gig workers’ behavior under algorithmic control by concurrently examining PSBs. Lastly, by revealing the moderating influence of WIC, we contribute to the theoretical boundaries of algorithmic control literature.

## 2. Theoretical Background

### 2.1. Affective Events Theory

The affective events theory suggests that important situations that occur in the workplace can trigger emotional reactions among employees, ultimately affecting their behavior and willingness [13]. Among them, workplace events, especially important events, can be summarized as work events. The affective events theory has been widely applied in various work scenarios [14], but there is still limited exploration regarding the unique and scattered distribution of employees in the gig economy.

Unlike traditional industries, for gig workers, algorithmic control often replaces the management of real supervisors [5], so algorithmic control can also be a typical work event for gig workers. Meanwhile, due to the duality of algorithm evaluation by gig workers, challenge and hindrance assessments can be considered as positive and negative events, respectively. Based on the affective events theory, these two different evaluations have varying degrees of impact on employee emotions and ultimately affect their prosocial service behaviors.

### 2.2. Challenge–Hindrance Stressor Framework and Challenge–Hindrance Appraisal Framework

Cavanaugh et al. [15] introduced the challenge–hindrance stressor framework (CHSF), classifying stressors based on whether they aid or impede employees’ goals. Challenge and hindrance appraisals elucidate the processes underpinning the effects of stressors, thereby enhancing the predictive validity of the CHSF. Initially proffered by Webster et al. [16], Searle and Auton [17] subsequently validated new measures of challenge and hindrance appraisals, showcasing their utility in stress research and articulating a distinct CHAF. Many empirical studies have since adopted this framework to explore the effects of AI [18,19], algorithm management [12,20], employee demands [21,22,23] and other factors on workers’ behaviors and intentions.

Platform algorithm control, recognized as a stressor, can be concurrently appraised as being either challenging or hindering, contingent upon its inherent nature and consequential effect [17]. This implies that stress may manifest with either beneficial or detrimental outcomes, subject to the manner in which employees appraise it [24]. Through the lens of the CHAF, it is posited that a challenge appraisal towards platform algorithm control will catalyze gig workers’ service behavior, whilst a hindrance appraisal will act to impede it.

### 2.3. Prosocial Service Behaviors

Prosocial service behaviors (PSBs) refer to helpful actions taken by customer-facing employees towards colleagues or customers. These behaviors encompass the following three dimensions: role-prescribed service behavior (RSB), extra-role service behavior (ESB) and cooperation [25]. RSB involves mandatory, standard behaviors expected by one’s role and organization. In contrast, ESB involves discretionary, spontaneous actions beyond formal role duties. Cooperation includes collaborative efforts with coworkers to improve service quality and efficiency.

PSBs are considered to be among the elements that can improve service quality and therefore have attracted the attention of service industry managers and scholars [25,26]. In the past, PSBs were mainly studied in traditional industries, such as tourism and the hospitality industry [26,27,28] and hospital industry [29,30]. Meanwhile, as mentioned earlier, PSBs have three dimensions, but currently some studies only use two dimensions [31,32]. In the gig economy, where tasks are independent and platform-assigned, this two-dimensional approach is adopted, omitting cooperation due to its reduced relevance in this context.

### 2.4. Workplace Interpersonal Capitalization

Watkins [33] delineated workplace interpersonal capitalization (WIC) as a scenario in which employees are predisposed to sharing personal, work-related positive events with their colleagues. The concept of WIC is relatively new, with only four empirical studies to date. Three of these studies focus on WIC’s impact on employees. Initially, Watkins [33] introduced the concept, highlighting responder-based reactions and identifying competition as a key factor influencing capitalization effects. Subsequently, Watkins et al. [34] then integrated capitalization theory with the social-functionalist model of emotions, developing a model that explains how WIC promotes knowledge sharing. Li et al. [14] explored how coworkers’ WIC correlates with employees’ contact avoidance through anxiety. These studies indicate that WIC has both positive and negative effects on employees’ emotions and behaviors. At the same time, there is also a literature study on the antecedent variable of WIC [35] which explored the impact mechanism and boundary effect of narcissism on WIC. Despite existing research that substantiates the initial reliability of WIC, the contexts in which it has been examined remain notably limited. Consequently, an expanded breadth of research on WIC is required.

## 3. Research Hypotheses

### 3.1. Algorithmic Control Appraisal and Gig Workers’ Prosocial Service Behaviors

Concomitant with the evolution of the gig economy, online labor platforms leverage algorithmic technology and data analytics to facilitate the swift and precise matching of labor supply and demand, rooted in digital technology [2]. For gig workers, the online labor platform algorithm conveys information related to platform rules, which actually contain specific requirements. These requirements are mandatory, which objectively promotes gig workers’ work behavior whether they are willing or not. In this way, mandatory algorithmic control may bring stress to gig workers. For example, China’s Meituan platform often requires delivery riders to deliver takeout within the specified time, which can result in penalties for exceeding the time limit, causing stress on many riders. The resultant work stress experienced by gig workers can wield either positive or negative impacts on their occupational outcomes, contingent upon the appraisal of the stressors [15].

Certain attributes of online labor platforms may prompt gig workers to render a challenge appraisal towards algorithmic control. The algorithmic technology, acting as a technological adjunct to assist gig workers, facilitates the pursuit of established work objectives and the acquisition of corresponding and additional rewards [36]. This implies that the pressure derived from algorithmic control concurrently ushers in potential advantages. Consequently, algorithmic control is perceived as challenging when algorithms categorize workers based on their service level to determine bonus incentives [37]. In this context, algorithmic control can also be construed as a motivational catalyst, propelling gig workers to fulfill the obligations and responsibilities intrinsic to their designated work role in exchange for platform rewards [8]. When the material rewards and positive emotional responses elicited by such challenges are sufficiently abundant, gig workers might also extend additional effort, confer extra benefits, and sporadically deliver exceptional services to attain a higher service level. Consequently, we posit that a challenging appraisal toward algorithmic control can catalyze gig workers’ PSBs. Thus, we propose that:

**H1a.**  *Challenge appraisals in regard to platform algorithm control positively predict gig workers’ role-prescribed service behavior.*

**H1b.**  *Challenge appraisals in regard to platform algorithm control positively predict gig workers’ extra-role service behavior.*

Moreover, specific attributes of the online labor platform can also prompt gig workers to formulate a hindrance appraisal in relation to algorithmic control. A hindrance appraisal directed toward platform algorithm control predominantly emanates from two aspects. Firstly, in light of the uncertain and real-time updated nature of algorithmic task assignments, gig workers frequently remain online for extensive periods to avert missing orders, considerably encroaching upon their personal lives and inducing burnout and anxiety [38,39]. This mental state potentially propels work inaccuracies and hinders gig workers from ensuring even the basic fulfillment of job prerequisites and their designated work role. Secondly, platform algorithms often penalize gig workers based on customer reviews, which do not require justifications, thereby cultivating an environment of perceived unfair treatment among platform workers [40]. Given such a customer-centric platform management policy, algorithmic control may inflict physical and psychological health issues on gig workers [5]. Consequently, it diminishes gig workers’ zeal and faith in customers, prompting them to expedite service completion, minimize communication with customers, and abstain from extending additional service behaviors toward them. Thus, we propose that:

**H2a.**  *Hindrance appraisals in regard to platform algorithm control negatively predict gig workers’ role-prescribed service behavior.*

**H2b.**  *Hindrance appraisals in regard to platform algorithm control negatively predict gig workers’ extra-role service behavior.*

### 3.2. The Mediating Mechanism of Work Engagement

Numerous scholarly investigations into the antecedents of work engagement have posited that work engagement is predominantly propelled by an amalgamation of job resources such as development opportunities, which are always positive work events in the affective events theory [11]. Given that challenge stressors afford opportunities for growth and development, they can be conceptualized as job resources [41]. Consequently, it is posited that a challenging appraisal in regard to algorithmic control can act as a positive work event, propelling gig workers towards heightened work engagement. Meanwhile, according to the affective events theory, hindrance stressors are typically inversely associated with work engagement, stemming from the notion that the exertion to surmount such stressors might generate negative emotions, thereby inflicting detrimental impacts on work engagement [41]. Consequently, it can be posited that hindrance appraisal toward algorithmic control may attenuate gig workers’ work engagement.

Concurrently, it has been observed that employees demonstrating pronounced levels of work engagement tend to foster a robust sense of identification with their occupational roles, thereby engaging in an expanded spectrum of tasks accompanied by a more positive emotional involvement [42]. Bakker [43] propounds that employees who exhibit engagement within their roles are predisposed to outperform their disengaged counterparts in terms of exertion and task accomplishment. Cheng and Chen [11] further underscore that engaged frontline employees not only exhibit a willingness to surpass formal role expectations in a bid to satiate client requirements but also strategically allocate their time and energy to optimize performance. Therefore, we argue that gig workers’ WE can positively lead to their PSBs, including RSB and ESB. In conclusion, we propose that:

**H3a.**  *Challenge appraisals in regard to platform algorithm control positively predict gig workers’ role-prescribed service behavior by increasing their work engagement.*

**H3b.**  *Challenge appraisals in regard to platform algorithm control positively predict gig workers’ extra-role service behavior by increasing their work engagement.*

**H4a.**  *Hindrance appraisals in regard to platform algorithm control negatively predict gig workers’ role-prescribed service behavior by decreasing their work engagement.*

**H4b.**  *Hindrance appraisals in regard to platform algorithm control negatively predict gig workers’ extra-role service behavior by decreasing their work engagement.*

### 3.3. The Moderating Role of Workplace Interpersonal Capitalization

Watkins [33] delineated the scenario in which employees are predisposed to sharing personal, work-related positive events with their colleagues, a phenomenon dubbed as WIC. We mainly focus on the moderating effect of WIC on the impact pathway related to gig worker’s work engagement, as the impact of capitalization is often related to employee emotions rather than directly affecting behavior [33,34]. The realization of positive outcomes through capitalization is contingent upon the degree of competition among respondents. In contexts where competition is minimal, capitalization tends to yield advantageous outcomes [33]. Gig workers, who fulfill service requests via platforms not constrained by the traditional parameters of fixed hours or locations [44], operate within an ecosystem that is algorithmically regulated and less competitive vis à vis peer interactions. This low-competition environment is conducive to the positive effects of capitalization.

As delineated by Smith [45] and Watkins [33], capitalization can bring “enhanced expectations for the future and a positive redefining of one’s capabilities”, which is precipitated by the superior example set by another individual. Within the framework of WIC, the presence of such feelings leads gig workers to harbor a more optimistic view of their future, conceptualizing it as being brighter and more promising upon successfully navigating through challenging stressors. Concurrently, this process significantly mitigates the effects of hindrance stressors. This motivational surge not only amplifies the positive impact of challenge appraisals directed at the algorithmic controls governing gig work platforms but also diminishes the detrimental influence of hindrance appraisals on these same algorithmic controls. Essentially, the inspiration derived from capitalization acts as a buffer, reshaping the gig workers’ response to environmental stressors and their interaction with the platform’s regulatory mechanisms, thereby fostering a more constructive engagement and adaptive coping strategies in the face of occupational challenges. Thus, we purpose that:

**H5a.**  *Workplace interpersonal capitalization significantly strengthens the relationship between challenge appraisals in regard to platform algorithm control and gig workers’ work engagement.*

**H5b.**  *Workplace interpersonal capitalization significantly weakens the relationship between hindrance appraisals in regard to platform algorithm control and gig workers’ work engagement.*

Furthermore, in an environment characterized by minimal competition, the success of a coworker can be perceived as mutually beneficial, fostering a sense of collective achievement among employees [33]. Within this context, the capitalization process facilitates a shared experience of positive events, consequently leading responders to perceive the discloser’s success as being within their potential reach. This perception is particularly pronounced under the auspices of workplace interpersonal capitalization, where gig workers are inclined to conceive greater opportunities for personal benefits or professional development.

This outlook, in turn, propels their work engagement, reinforcing the positive feedback loop that enhances their commitment and vigor towards work. Therefore, in the unique dynamic of the gig economy, where traditional competitive structures are often diluted, the positive repercussions of a peer’s success through capitalization can resonate more deeply with individuals, incentivizing them and bolstering their engagement with the work at hand. The ensuing sense of attainable success and personal growth not only elevates the individual’s work engagement but also fortifies the overall positive impact of such engagement within the gig work environment. Therefore, we purpose the following:

**H6a.**  *Workplace interpersonal capitalization significantly strengthens the relationship between work engagement and gig workers’ role-prescribed service behavior.*

**H6b.**  *Workplace interpersonal capitalization significantly strengthens the relationship between work engagement and gig workers’ extra-role service behavior.*

**Figure 1:**
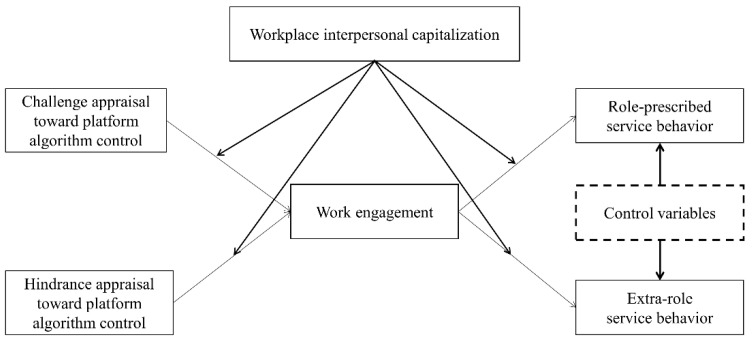
Research framework.

In summary, we propose the research framework in Figure 1, as follows:

## 4. Methodology

### 4.1. Data Collection

In this study, the research participants are platform-based gig workers, including online taxi drivers and food delivery personnel. Given the extensive spatial dispersion of this workforce, traditional offline methods of data collection, such as face-to-face interviews, are impractical. Therefore, we utilized an online questionnaire distribution as our data collection strategy to gather the necessary information to test our research hypotheses.

We conducted an online survey in September 2023 to assess our hypotheses, employing questionnaires as our data collection instrument. The survey data were gathered through the Credamo platform, which is a leading online data collection platform in China and has been widely adopted to collect questionnaire data in the previous literature [46,47]. With a compensation of around CNY 3–5, we can provide questionnaires to designated gig workers through Credamo. Out of an initial 700 respondents, we excluded some questionnaires with identical answers throughout or with obvious contradictions in responses, resulting in 660 valid questionnaires for empirical analysis. The demographic characteristics of the participant sample, delineated in Table 1, reveal particular distributions across several variables. Specifically, a majority, constituting 57.88%, of the respondents identify as male. Within the age dimension, the predominant age bracket encompasses those aged 31–40, comprising 43.94% of the total participants. In terms of educational attainment, a significant majority, at 96.21%, are reportedly without an undergraduate degree. Considering professional experience, 62.72% of the participants possess a work history extending beyond two years. Moreover, a noteworthy 67.11% attest to engaging in work-related activities for a duration exceeding four hours daily.

### 4.2. Measurement

The items for measurement are from the existing literature (see Appendix A). The measurement items of challenge (CTA) and hindrance (HTA) appraisals in regard to platform algorithm control applied in this study refer to Searle and Auton [17] and Ding [20]. The items measuring the constructs of work engagement (WE) were proposed by Schaufeli et al. [48] and Qin et al. [49]. The measurement items for prosocial service behaviors (PSBs), comprising role-prescribed service behavior (RSB) and extra-role service behavior (ESB), were adapted from Tsaur et al. [26]. Finally, the items measuring the constructs of workplace interpersonal capitalization (WIC) were proposed by Watkins [33]. All constructs were assessed using a Likert five-point scale where one corresponds to “strongly disagree” and five denotes “strongly agree”, with higher numbers indicating greater agreement. The questionnaires were submitted to experts for review, and the initial questionnaire was then refined based on the experts’ feedback.

## 5. Results

### 5.1. Common Method Deviation Test

As the survey responses are based on individuals’ self-reported data in a single questionnaire, we first applied Harman’s single-factor approach to detect any common method bias [50]. The result indicates that the first and largest factor explains about 39.70% of the covariance among our main measures, falling below the critical 50% threshold. Second, we employed the latent error variable control method [51], introducing common method bias as a latent variable in the model for further examination [52]. After incorporating the latent variable, the changes were as follows: ΔRMSEA = 0.008, ΔGFI = 0.033, ΔCFI = 0.049, and ΔTLI = 0.043. These changes were not substantial (Δ < 0.05), indicating that the paper does not suffer from significant common method bias.

### 5.2. Reliability Testing

We evaluated the measurement model to assess the validity and reliability of the constructs, which are shown in Table 2. Notably, all item loadings and Cronbach’s alpha values surpass 0.7, while composite reliabilities (CR) values and average variance extracted (AVE) values are all greater than 0.7. These results, aligned with the criteria established by Fornell and Larcker [53], indicate a high degree of validity and reliability for the constructs.

### 5.3. Correlation Analysis

Table 3 illustrates the findings on discriminant validity, achieved by comparing the square root of each construct’s AVE with the corresponding correlation coefficients. The results in Table 3 show that the square roots of the AVEs range from 0.80 to 0.89, and the largest correlation is 0.52 between WE and CTA, demonstrating strong discriminant validity. Meanwhile, to address potential multicollinearity issues arising from some large correlations, we conducted a variance inflation factor (VIF) test. The results indicated that the highest observed VIF value was below 2, suggesting no significant multicollinearity concerns [50,54].

### 5.4. Structural Model

To estimate the regression coefficients, STATA 17 software was employed. Additionally, understanding that viewers are not a homogeneous group and that socio-demographic factors can influence their behaviors and perceptions, we controlled the effects of gender, age, education, professional experience, and working hours. The primary results are presented in Table 4, which showed that CTA positively affected both RSB (0.31, *p* < 0.01) and ESB (0.32, *p* < 0.01). Therefore, H1a and H1b were supported. In addition, HTA negatively affected RSB (−0.27, *p* < 0.01) and ESB (−0.30, *p* < 0.01), supporting H2a and H2b.

The mediation effect of WE was tested using the process outlined by Luo et al. [50]. The results are presented in Table 5. In the first step, we examined the effects of CTA and HTA on WE. We found significant effects of both CTA (0.30, *p* < 0.01) and HTA (−0.16, *p* < 0.01). Secondly, we incorporated CTA and WE in a single regression to investigate their effects on RSB and ESB. The results showed that WE significantly and positively influenced RSB (0.22, *p* < 0.01) and ESB (0.24, *p* < 0.01); concurrently, the coefficients of CTA decreased to 0.22, a figure notably inferior to both 0.31 and 0.32, as presented in Table 4, when solely scrutinizing the impact of CTA on RSB and ESB, respectively. Similarly, we also incorporated HTA and WE in a single regression to investigate their effects on RSB and ESB, finding that the effect of WE was significant and positive (RSB: 0.25, *p* < 0.01; ESB: 0.26, *p* < 0.01); however, the coefficients corresponding to HTA dwindled to −0.19 and −0.22, metrics that are manifestly lower than −0.27 and −0.30, as specified in Table 4. Lastly, a comprehensive regression, amalgamating CTA, HTA, and WE, was deployed to delineate their collective effects on RSB and ESB, yielding significant coefficients across all three variables. It is imperative to note that, throughout all three investigative phases, the coefficients corresponding to CTA and HTA were consistently inferior when compared to the values enumerated in Table 4. Thus, WE partially mediates the relationships between CTA, HTA, and gig workers’ PSBs. Therefore, H3a, H3b, H4a, and H4b are supported.

Further, we examined the moderation effect of WIC on the relationships between both CTA and HTA with WE and between WE with RSB and ESB. The results are shown in Table 6. In the first analysis, WIC significantly and positively moderated the relationship between CTA and WE (0.10, *p* < 0.01), supporting H5a. On the other hand, the moderation effect of WIC was not significant in the relationship between HTA and WE (−0.02, *p* > 0.1), so H5b is not supported. Finally, WIC strongly promote the influence of WE on RSB (0.11, *p* < 0.01) and ESB (0.10, *p* < 0.01). Accordingly, H6a and H6c are supported.

### 5.5. Bootstrap Check

With the bootstrap method, we further investigated the mediating effects in our study. Table 7 shows the results of the serial mediation analysis for all possible pathways between algorithmic control appraisals and gig workers’ PSBs using model four of Hayes’ PROCESS bootstrapping mediation [55]. The results reveal that the pathways from CTA and HTA to RSB and ESB are mediated by WE. Specifically, as shown in Table 6, the indirect effect of CTA on RSB via WE is significant and positive (0.0939, 95% CI [0.0547, 0.1358]) and the indirect effect of CTA on ESB through WE is also significant and positive (0.1019, 95% CI [0.0605, 0.1484]). Meanwhile, the indirect effect of HTA on RSB through WE is significant and negative (−0.0738, 95% CI [−0.1058, −0.0464]), and the indirect effect of HTA on ESB through WE is also significant and negative (−0.0758, 95% CI [−0.1080, −0.0467]). These findings provide robust evidence of the significant mediating role of WE, reaffirming the validity of our primary results.

## 6. Discussion and Implications

Employing the STATA data processing tool, bootstrap methodology and structural equation modeling (SEM), we discerned several crucial insights. While most of the proposed hypotheses were substantiated, one particular hypothesis—that workplace interpersonal capitalization significantly mitigates the relationship between hindrance appraisal and work engagement (H5b)—was not confirmed. One potential rationale is that, confronted with a high hindrance appraisal of platform algorithm control, customers might develop a stereotypical perception of the platform algorithm, leading them to execute tasks assigned by the algorithm mechanically. This lack of expectation for algorithmic control prompts them to disregard a substantial amount of external information, potentially diminishing the effectiveness of workplace interpersonal capitalization. In conclusion, irrespective of whether the hypotheses were confirmed or refuted, the findings of this study yield significant implications. By applying the challenge–hindrance appraisal framework, prosocial service behaviors, and workplace interpersonal capitalization in the field of algorithmic control in the gig economy, we have expanded the theoretical boundaries of several concepts and deepened our understanding of algorithmic control in the gig economy, resulting in theoretical and practical contributions.

### 6.1. Theoretical Implications

Firstly, this study augments the discourse in the algorithm control literature, presenting a nuanced understanding of the dual impacts of algorithm control on gig workers. While burgeoning research explores algorithmic control’s effects on this sector, findings thus far have been contradictory. Existing studies illustrate that algorithmic control can yield both positive [56] and negative [57,58] outcomes for employees, yet they fall short of providing an intuitive and comprehensive framework to articulate this duality effectively. Through the incorporation of the challenge–hindrance appraisal framework, this paper posits that the divergences in gig workers’ perceptions of algorithmic control precipitate the variance in their emotional and behavioral repercussions. The construction of this dual-path model furnishes a more dialectical theoretical paradigm for interpreting the function of algorithmic control. Furthermore, the research emphasizes the challenge–hindrance appraisal framework’s versatility and applicability in new and evolving contexts, thereby expanding its theoretical and practical utility.

Secondly, this study aims to investigate the impact of algorithmic control on gig workers’ prosocial service behaviors, embodying an interdisciplinary approach that spans organizational behavior, psychology, and technology management. By refining the research findings, we aim to enhance our comprehensive understanding of human behavior, particularly within digital work environments. This research identifies and substantiates novel elements that influence employees’ prosocial service behaviors, thereby broadening their applicability to new environments. The previous literature has thoroughly investigated the antecedent variables influencing employees’ prosocial service behaviors from the vantage point of role stress [28], leadership [30,32], management strategies [59], and job resourcefulness [11]. However, within the burgeoning domain of online labor platforms, where algorithms are central, these platforms introduce a new dynamic in the employee interaction sphere. The empirical evaluation of the algorithm’s impact on employees remains relatively nascent in current scholarly works. By employing the challenge–hindrance appraisal framework and the mediating role of work engagement, this study concurrently assesses the enabling and obstructive effects of algorithmic control on employees’ prosocial service behavior. This approach not only enriches the literature concerning the antecedent variables of prosocial service behavior but also provides an innovative perspective in understanding employee interactions and behaviors in algorithm-driven work environments.

Lastly, this paper significantly enhances our understanding of algorithmic control by delving into its boundary conditions. The preponderance of extant research predominantly centers on the intrinsic aspects of algorithmic control and the influence of employee traits on behavior while largely overlooking the ramifications of social influences external to the algorithms themselves. In an innovative approach, we integrate workplace interpersonal capitalization as a moderating variable, examining its influence on the relationship between gig workers’ appraisals of algorithmic control and their work engagement as well as between their work engagement and subsequent prosocial service behaviors. Our findings suggest that heightened workplace interpersonal capitalization exerts a favorable regulatory impact on gig workers’ social service behaviors. Thus, from this analytical standpoint, workplace interpersonal capitalization emerges as a critical theoretical boundary in the exploration of the multifaceted effects of algorithmic control.

### 6.2. Practical Implications

Our research bears significant practical implications for entities intending to oversee employees via algorithm control. Initially, our study found that challenge appraisal of platform algorithmic control can positively influence gig workers’ role-prescribed service behavior and extra-role service behavior. However, hindrance appraisal of platform algorithmic control can negatively influence gig workers’ role-prescribed service behavior and extra-role service behavior. It is crucial to recognize the dual nature of algorithmic control evaluations. Therefore, appraisals in regard to algorithm control become prudent for these organizations in amplifying the perception of algorithm control as a challenge, circumventing its interpretation as a hindrance. These organizations should carefully guide gig workers to view algorithmic control as a challenge and avoid interpreting it as a hindrance. It is necessary for gig workers to comprehend that a “challenge” inherently conveys the notion of potential rewards following the surmounting of difficulties. Consequently, platforms can organize training seminars to explain how algorithmic control operates, its purpose, and how it boosts efficiency and service quality, thereby improving gig workers’ perceptions of it. Additionally, integrating gamification elements, like challenges, leaderboards, and rewards, can enhance employees’ view of algorithmic control as a stimulating challenge.

Furthermore, workplace interpersonal capitalization not only positively affects the relationship between challenge appraisals in regard to platform algorithm control and gig workers’ work engagement but also significantly strengthens the relationship between work engagement and gig workers’ prosocial service behaviors. The pronounced moderating impact of workplace interpersonal capitalization underscores the value of employee communities that exist independently of algorithmic frameworks. Given that employees’ self-initiated private communities elude official oversight, platforms could consider formally instituting a cohesive work community for more structured management. Within this community, disseminating narratives of successful employees and highlighting affirmative events could serve as a potent motivational force for employees, fostering a more engaged and productive workforce.

Concurrently, it is imperative to recognize that workplace interpersonal capitalization fails to diminish the detrimental effects of hindrance evaluations on employees’ prosocial service behaviors. This ancillary confirmation underscores the necessity for platform managers to prevent employees’ hindrance evaluations in regard to algorithm control. Once these negative assessments are formed, they prove resistant to alteration through supplementary interventions. This highlights the urgent need for proactive measures aimed at reconfiguring perceptions of algorithmic control, preventing them from being construed as hindrances and thus creating an environment more supportive of positive employee outcomes and engagement. Platforms may contemplate enhancing transparency by presenting employees with select logic and data employed in algorithmic decision-makin while ensuring the safeguarding of proprietary information. This approach facilitates employees’ comprehension that algorithms are not opaque “black boxes”. Additionally, the establishment of a designated feedback avenue is essential for enabling employees to offer their perspectives and proposals regarding algorithmic governance at their convenience. This ensures that gig workers do not perceive algorithmic control as an impediment.

## 7. Conclusions

Based on the affective events theory, our research rigorously investigates the factors influencing gig workers’ PSBs within the context of online labor platforms, employing CHAF to address the pivotal role of algorithmic control. Utilizing an online questionnaire survey, we meticulously gathered empirical data to thoroughly test our hypotheses. Our findings reveal the complex dual impact of challenge–hindrance appraisals in regard to algorithmic control over PSBs among gig workers. This relationship is elucidated through the mediating role of WE and the moderating influence of WIC. The contributions of our research are multifaceted. Firstly, by applying CHAF, we enhance the understanding of the nuanced duality inherent in algorithmic control. This insight suggests that managers should focus on fostering employees’ perceptions of challenges rather than hindrances. Secondly, the examination of PSBs underscores their applicability across multiple industries. This not only extends the theoretical scope of PSBs but also equips managers in various sectors with insights into industry interconnections and sustainable development strategies. Finally, the exploration of WIC not only validates the practicality of this concept but also broadens the theoretical understanding of algorithmic control. It further provides managers with critical perspectives on the determinants of employee service behavior that extend beyond the sole realm of algorithmic control.

## 8. Limitations and Future Research Directions

Our research, while insightful, is not exempt from limitations. Primarily, the use of a questionnaire survey method for data collection introduces an element of subjectivity, as respondents’ perceptions may be swayed by their immediate environment, emotional state, and various other extraneous variables. Subsequent research could enhance the robustness of these findings by incorporating qualitative methodologies, such as comprehensive interviews, to corroborate the data’s reliability. Additionally, the study focused primarily on temporary workers in China, which may have limited the generalizability of the findings. Future studies should include more diverse geographic contexts or compare findings across regions.

Furthermore, our choice of work engagement as the sole mediator, owing to its well-documented correlation with prosocial service behaviors and challenge–hindrance appraisals, might be perceived as lacking in diversity. Given that hindrance appraisals can precipitate negative emotional and cognitive responses, future research would do well to integrate and explore negative mediating factors.

In addition, in assessing the influence of the communities within which gig workers operate, we opted for the subjective construct of workplace interpersonal capitalization. It is imperative to recognize, however, that gig workers’ communities are often diffuse and can encompass intersecting micro-communities. Therefore, an objective assessment is urgently required to discern the effects of these unique communal relationships on gig workers, thus providing a more nuanced understanding of their experiences and behaviors.

## Figures and Tables

**Table 1:** Sample demographic.

Characteristics	Levels	Frequency	Percentage (%)
Gender (GE)	Male	382	57.88
	Female	278	42.12
Age group (AG)	<30	158	23.94
	31–40	290	43.94
	41–50	187	28.33
	>51	25	3.79
Education (EDU)	≤primary school	24	3.64
	junior school	216	32.73
	high school	149	22.58
	junior college	246	37.27
	≥undergraduate	25	3.79
Professional Experience (PE)	≤1 year	82	12.42
	1–2 years	164	24.85
	2–3 years	224	33.94
	3–5 years	141	21.36
	≥5 years	49	7.42
Work time (TI)	0–1 h	7	1.06
	1–4 h a day	210	31.82
	4–7 h a day	180	27.27
	7–10 h a day	187	28.33
	10–13 h a day	68	10.3
	≥13 h a day	8	1.21

**Table 2:** Scale properties.

	Items	Factor Loading	Cronbach’s Alpha	CR	AVE
Challenge appraisal toward platform algorithm control (CTA)	CTA1	0.90	0.84	0.90	0.75
	CTA2	0.86			
	CTA3	0.85			
Hindrance appraisal toward platform algorithm control (HTA)	HPA1	0.92	0.87	0.92	0.79
	HPA2	0.87			
	HPA3	0.89			
Work engagement (WE)	WE1	0.83	0.72	0.84	0.64
	WE2	0.84			
	WE3	0.74			
Role-prescribed service behavior (RSB)	RSB1	0.90	0.81	0.89	0.72
	RSB2	0.77			
	RSB3	0.87			
Extra-role Service behavior (ESB)	ESB1	0.88	0.79	0.88	0.71
	ESB2	0.75			
	ESB3	0.88			
Workplace interpersonal capitalization (WIC)	WIC1	0.91	0.92	0.94	0.76
	WIC2	0.84			
	WIC3	0.91			
	WIC4	0.81			
	WIC5	0.89			

**Table 3:** Correlations and discriminant validity.

	CTA	HTA	WE	RSB	ESB	WIC	GE	AG	EDU	PE	TI
CTA	0.87										
HTA	−0.44 **	0.89									
WE	0.52 **	−0.40 **	0.80								
RSB	0.41 **	−0.37 **	0.40 **	0.85							
ESB	0.36 **	−0.36 **	0.37 **	0.72 **	0.84						
WIC	0.52 **	−0.35 **	0.43 **	0.46 **	0.33 **	0.87					
GE	0.15 **	−0.05	0.11 **	−0.01	0.01	0.09 *	--				
AG	−0.01	0.00	−0.02	−0.15 **	−0.01	−0.10 *	0.35 **	--			
EDU	0.29 **	−0.17 **	0.22 **	0.17 **	0.07	0.26 **	0.47 **	0.17 **	--		
PE	0.31 **	−0.25 **	0.29 **	0.27 **	0.23 **	0.28 **	0.29 **	0.32 **	0.43 **	--	
TI	0.17 **	−0.10 **	0.17 **	0.07	0.14 **	0.10 **	0.36 **	0.35 **	0.30 **	0.47 **	--

Note: * *p* < 0.05, ** *p* < 0.01. Two-tailed test. The diagonally arranged data are the square roots of AVEs.

**Table 4:** Estimation results 1.

	RSB	ESB	RSB	ESB
CTA	0.31 ***	0.32 ***		
	(0.03)	(0.04)		
HTA			−0.27 ***	−0.30 ***
			(0.03)	(0.04)
GE	−0.15 *	−0.11	−0.11	−0.06
	(0.08)	(0.09)	(0.08)	(0.09)
AG	−0.24 ***	−0.07	−0.26 ***	−0.09 *
	(0.04)	(0.05)	(0.05)	(0.05)
EDU	0.04	−0.07 *	0.07 *	−0.05
	(0.04)	(0.04)	(0.04)	(0.04)
PE	0.20 ***	0.14 ***	0.22 ***	0.15 ***
	(0.04)	(0.04)	(0.04)	(0.04)
TI	−0.01	0.07 *	0.00	0.08 *
	(0.04)	(0.04)	(0.04)	(0.04)
Constant	2.67 ***	2.34 ***	4.27 ***	4.06 ***
	(0.16)	(0.18)	(0.17)	(0.18)
Observations	660	660	660	660
Adj-R2	0.24	0.15	0.22	0.15
F	34.83	20.58	32.06	20.90
RMSE	0.83	0.93	0.84	0.92

Note: *** *p* < 0.01, * *p* < 0.1.

**Table 5:** Estimation results 2.

	WE	RSB	ESB	RSB	ESB	RSB	ESB
CTA	0.35 ***	0.22 ***	0.22 ***			0.17 ***	0.16 ***
	(0.03)	(0.04)	(0.04)			(0.04)	(0.04)
HTA	−0.16 ***			−0.19 ***	−0.22 ***	−0.15 ***	−0.19 ***
	(0.03)			(0.03)	(0.04)	(0.03)	(0.04)
WE		0.22 ***	0.24 ***	0.25 ***	0.26 ***	0.19 ***	0.20 ***
		(0.04)	(0.05)	(0.04)	(0.04)	(0.04)	(0.05)
GE	0.01	−0.15 *	−0.10	−0.12	−0.07	−0.13 *	−0.08
	(0.07)	(0.08)	(0.09)	(0.08)	(0.09)	(0.08)	(0.08)
AG	−0.08 **	−0.22 ***	−0.05	−0.23 ***	−0.06	−0.22 ***	−0.04
	(0.04)	(0.04)	(0.05)	(0.04)	(0.05)	(0.04)	(0.05)
EDU	0.02	0.04	−0.08 *	0.05	−0.07	0.03	−0.09 **
	(0.04)	(0.04)	(0.04)	(0.04)	(0.04)	(0.04)	(0.04)
PE	0.09 ***	0.18 ***	0.11 ***	0.18 ***	0.11 ***	0.16 ***	0.09 **
	(0.03)	(0.04)	(0.04)	(0.04)	(0.04)	(0.04)	(0.04)
TI	0.04	−0.02	0.06	−0.01	0.06	−0.02	0.06
	(0.03)	(0.04)	(0.04)	(0.04)	(0.04)	(0.04)	(0.04)
Constant	2.50 ***	2.26 ***	1.89 ***	3.30 ***	3.07 ***	2.93 ***	2.72 ***
	(0.20)	(0.18)	(0.20)	(0.22)	(0.24)	(0.23)	(0.26)
Observations	660	660	660	660	660	660	660
Adj-R2	0.32	0.27	0.19	0.27	0.20	0.29	0.22
F	44.82	35.59	22.54	35.69	24.42	34.60	23.58
RMSE	0.77	0.81	0.91	0.81	0.90	0.80	0.89

Note: *** *p* < 0.01, ** *p* < 0.05, * *p* < 0.1.

**Table 6:** Estimation results 3.

	WE	WE	RSB	ESB
CTA	0.37 ***			
	(0.04)			
HTA		−0.23 ***		
		(0.03)		
WE			0.25 ***	0.30 ***
			(0.04)	(0.04)
WIC	0.18 ***	0.24 ***	0.27 ***	0.20 ***
	(0.03)	(0.03)	(0.03)	(0.04)
WIC*CTA	0.10 ***			
	(0.03)			
WIC*HTA		−0.02		
		(0.03)		
WIC*WE			0.11 ***	0.10 ***
			(0.03)	(0.04)
GE	0.02	0.04	−0.13 *	−0.08
	(0.07)	(0.08)	(0.08)	(0.09)
AG	−0.04	−0.07	−0.17 ***	−0.01
	(0.04)	(0.04)	(0.04)	(0.05)
EDU	0.01	0.03	0.03	−0.08 *
	(0.04)	(0.04)	(0.04)	(0.04)
PE	0.08 **	0.10 ***	0.14 ***	0.10 **
	(0.03)	(0.04)	(0.04)	(0.04)
TI	0.05	0.06	−0.01	0.07 *
	(0.03)	(0.04)	(0.03)	(0.04)
Constant	1.27 ***	2.90 ***	1.84 ***	1.61 ***
	(0.18)	(0.20)	(0.19)	(0.21)
Observations	660	660	660	660
Adj-R2	0.33	0.27	0.31	0.19
F	40.63	30.98	38.09	20.63
RMSE	0.77	0.80	0.79	0.90

Note: *** *p* < 0.01, ** *p* < 0.05, * *p* < 0.1.

**Table 7:** Bootstrap results for the mediation effect.

**Indirect impact of CTA on RSB**			
	Effect	Boot standard error	95% confidence interval
WE	0.0939	0.0207	[0.0547, 0.1358]
**Indirect impact of CTA on ESB**			
	Effect	Boot standard error	95% confidence interval
WE	0.1019	0.0225	[0.0605, 0.1484]
**Indirect impact of HTA on RSB**			
	Effect	Boot standard error	95% confidence interval
WE	−0.0738	0.0150	[−0.1058, −0.0464]
**Indirect impact of HTA on ESB**			
	Effect	Boot standard error	95% confidence interval
WE	−0.0758	0.0155	[−0.1080, −0.0467]

## Data Availability

The raw data supporting the conclusions of this article will be made available by the authors on request.

## Appendix A

Challenge appraisal toward platform algorithmic control (Ding, 2021 [20]; Searle and Auton, 2015 [17])
  1 Efforts to meet the requirements of the platform’s algorithm control help improve my personal growth and well-being.
  2 I feel that the requirements of the platform algorithm control challenge me to achieve my personal goals and achievements.
  3 Overall, I feel that the platform algorithmic control enhances my personal sense of accomplishment.
Hindrance appraisal toward platform algorithmic control (Ding, 2021 [20]; Searle and Auton, 2015 [17])
  1 Working to meet the demands of the platform’s algorithmic control hinders my personal growth and well-being.
  2 I feel that the requirement of platform algorithm control restricts the realization and development of my personal goals.
  3 Overall, I feel that platform algorithmic control reduces my personal sense of accomplishment.
Work engagement (Qin et al., 2018 [49]; Schaufeli et al., 2006 [48])
  1 I am immersed in my work
  2 When I am completely immersed in my work, I feel happy.
  3 I forget myself at work.
Role-prescribed service behavior (Tsaur et al., 2014 [26])
  1 I complete all the tasks requested by the customer.
  2 I help the customer handle the things he/she requests.
  3 I fulfill my responsibilities to customers as stipulated in the job description.
Extra-role service behavior (Tsaur et al., 2014 [26])
  1 I volunteer to assist customers even beyond work requirements.
  2 When serving customers, I often go above and beyond the call of duty.
  3 I am willing to do whatever it takes to keep my customers happy.
Workplace interpersonal capitalization (Watkins, 2021 [33])
  1 My colleagues in the gig economy routinely communicate their positive work experiences with me.
  2 We, as gig workers, collectively discuss favorable incidents that occur in our jobs.
  3 When fellow gig workers receive favorable work-related news, they readily share it with the group.
  4 It is common practice among my gig-working peers to inform the rest of us about their positive job occurrences.
  5 There is a frequent exchange of information about positive job-related events among the gig workers in my network.
